# Comparative genotypic and phenotypic analysis of human peripheral blood monocytes and surrogate monocyte-like cell lines commonly used in metabolic disease research

**DOI:** 10.1371/journal.pone.0197177

**Published:** 2018-05-10

**Authors:** Darren M. Riddy, Emily Goy, Philippe Delerive, Roger J. Summers, Patrick M. Sexton, Christopher J. Langmead

**Affiliations:** 1 Drug Discovery Biology, Monash Institute of Pharmaceutical Sciences, Monash University, Parkville, Victoria, Australia; 2 Institut de Recherches Servier, Pôle d’Innovation Thérapeutique Métabolisme, Suresnes, France (PD); Universitatsklinikum Freiburg, GERMANY

## Abstract

Monocyte-like cell lines (MCLCs), including THP-1, HL-60 and U-937 cells, are used routinely as surrogates for isolated human peripheral blood mononuclear cells (PBMCs). To systematically evaluate these immortalised cells and PBMCs as model systems to study inflammation relevant to the pathogenesis of type II diabetes and immuno-metabolism, we compared mRNA expression of inflammation-relevant genes, cell surface expression of cluster of differentiation (CD) markers, and chemotactic responses to inflammatory stimuli. Messenger RNA expression analysis suggested most genes were present at similar levels across all undifferentiated cells, though notably, *IDO1*, which encodes for indoleamine 2,3-dioxygenase and catabolises tryptophan to kynureninase (shown to be elevated in serum from diabetic patients), was not expressed in any PMA-treated MCLC, but present in GM-CSF-treated PBMCs. There was little overall difference in the pattern of expression of CD markers across all cells, though absolute expression levels varied considerably and the correlation between MCLCs and PBMCs was improved upon MCLC differentiation. Functionally, THP-1 and PBMCs migrated in response to chemoattractants in a transwell assay, with varying sensitivity to MCP-1, MIP-1α and LTB-4. However, despite similar gene and CD expression profiles, U-937 cells were functionally impaired as no migration was observed to any chemoattractant. Our analysis reveals that the MCLCs examined only partly replicate the genotypic and phenotypic properties of human PBMCs. To overcome such issues a universal differentiation protocol should be implemented for these cell lines, similar to those already used with isolated monocytes. Although not perfect, in our hands the THP-1 cells represent the closest, simplified surrogate model of PBMCs for study of inflammatory cell migration.

## Introduction

Monocyte recruitment and migration occurs as a consequence of conditioning of the local microenvironment by the release of microbial products, growth factors and pro-inflammatory mediators. Monocytes are able to differentiate into macrophages and dendritic cells (DC) when stimulated by different growth factors, including granulocyte-macrophage colony stimulating factor (GM-CSF) or macrophage colony stimulating factor (M-CSF), culminating in an effective controlling and clearing of the inflamed areas. In addition, differentiated cells can be further activated by various cytokines that result in their polarization [1, 2], yielding further release of pro-inflammatory cytokines and chemokines including TNF-α, IL-6, IL-1β and MCP-1 (CCL2) [3–5].

Recently, evidence has emerged from both animal models of disease and patients with insulin resistance and T2DM, that immune cell infiltration into adipose tissue causes chronic low-grade inflammation that plays a key role in the pathogenesis of obesity-induced insulin resistance [6–11]. An insight into the mechanistic link between insulin resistance and inflammation has revealed activation of various inflammatory signalling pathways and an increase in the expression of pro-inflammatory cytokines, including IL-6, TNF-α and IL-18 [12–14]. Furthermore, there is clear evidence demonstrating an increase in the number of proinflammatory macrophages present [15].

Monocyte like cell lines (MCLCs), including THP-1 and HL-60 cells (derived from patients with acute monocytic leukemia) and U-937 cells (immortalised from a patient with histiocytic lymphoma), are used routinely as surrogates for isolated CD14^+^ human peripheral blood mononuclear cells (PBMCs). These cell lines have been extensively characterised based on the mRNA expression levels of a selection of inflammatory mediators, including cytokines and chemokines [16–18], and in some cases used to monitor changes in expression levels following manipulation of specific gene targets [19]. Many researchers have utilised immortalised cell lines to help investigate the roles of immune cells in the development of metabolic diseases [19]. However, it is not clear to what extent these cell lines replicate the phenotype of PBMCs with respect to metabolic inflammation. Furthermore, in comparison to PBMCs, there is only limited information on the translation of mRNA levels to cellular function. In many cases only a single endpoint has been examined (e.g. cytokine release [20]) and typically only in polarized macrophages [20].

In the current study a comprehensive genotypic and phenotypic analysis of MCLCs compared to human CD14^+^ PBMCs was performed, investigating (a) expression of a panel of inflammatory genes (including both ligands and receptors); (b) the expression profile of cluster of differentiation (CD) markers by FACS and (c) the functional ability of the cells to migrate in response to various inflammatory stimuli. A further comparison was made between undifferentiated and differentiated monocytes (using either phorbol 12-myristate 13-acetate (PMA) treatment for the MCLCs or GM-CSF treatment for the PBMCs to establish a proinflammatory phenotype) [21]. PMA was preferred to other differentiation factors (e.g. DMSO and VitD3) as it is the most widely used for immortalised cell lines [18], and as both PMA and GM-CSF tend to generate a pro-inflammatory macrophage phenotype, it was our intention to determine just how reflective the former is of the latter. To assess differences upon macrophage polarisation, a further comparison was made between the GM-CSF-differentiated PBMCs and those activated with lipopolysaccharide (LPS) and interferon gamma (IFNγ).

Overall, most of the genes studied were expressed at similar levels across the MCLCs and PBMCs, but with some relevant and significant exceptions for genes involved in immune-metabolism (e.g. *KYNU*, *IL6*, *IL10*, *CCL4* and *IL1R2*), which is reflected in a hierarchical clustering analysis that clearly separates primary PBMCs from the monocyte-like cell lines.

The pattern of CD marker expression in THP-1 and U-937 cells was also similar to PBMCs, particularly under differentiated conditions that yield a macrophage/DC-like phenotype. The differentiated THP-1 cells, however, yield a “macrophage-only” phenotype as there was no expression of the DC markers CD80 or CD86. Both THP-1 and PBMCs migrated in response to the various chemoattractants using a transwell migration assay. However, surprisingly, U-937 cells failed to migrate to any chemoattractant, despite similar gene and CD expression profile compared to the other cell types. Collectively these data highlight the necessity for careful interpretation of data generated using these immortalized cell lines and the limitations on their utility as surrogates for human PBMCs especially when used to further understand the role of immune cells in the development and progression of metabolic diseases.

## Materials and methods

### Cell line maintenance, CD14^+^ PBMC isolation, culture and differentiation

Monocyte-like cell lines U-937, THP-1 and HL-60s were a generous gift from Dr. Nick Huntington from the Walter and Eliza Hall Institute of Medical Research, Parkville, Australia. The cell lines were cultured in RPMI-1640 growth medium (Thermo Fisher Scientific, Scoresby) supplemented with 10% v/v fetal bovine serum (FBS; Thermo Fisher Scientific, Scoresby) and incubated at 37°C with 5% CO_2_. Differentiation of these cells into a macrophage-like phenotype was by treatment with 16 ng/mL phorbol-12-myristate-13-acetate (PMA; Sigma-Aldrich, Castle Hill) for 48 h. Following treatment, differentiated cells were washed twice with PBS before being dissociated with Accutase solution (Sigma-Aldrich, Castle Hill). All cell lines were shown to be absent of mycoplasma or admixture contamination during this study.

Collection and use of human blood samples (buffy coat and whole blood) was conducted according to the guidelines and approval of Monash University (Clayton, Australia) and the Monash University Research Ethics Committee (Clayton, Australia). Blood was obtained from either 100 mL donation of whole blood obtained by venepuncture of consenting human volunteers from the Victorian Blood Donor Registry or from buffy coat preparations from the Australian Red Cross blood service (Melbourne, Australia, under the Monash University Research Ethics Committee approval; HREC CF14/999e2014000425). Blood was collected by written consent and was used by the Blood Service or other organisations for the purposes of research.

CD14^+^ PBMCs were isolated from blood buffy coat from human donor samples using a Ficoll gradient and CD14^+^ magnetic bead separation method (Miltenyi Biotech, Germany). Cells were cultured in α-MEM (Thermo Fisher Scientific, Scoresby) supplemented with 10% FBS (v/v) and 1% penicillin streptomycin (v/v; Thermo Fisher Scientific, Scoresby). PBMCs were differentiated using 10 ng/mL granulocyte macrophage colony-stimulating factor (GM-CSF; R&D Systems, Minneapolis, US) for 6 days. Differentiated cells were washed and dissociated as described above. GM-CSF differentiated proinflammatory macrophages were activated using 100 ng/mL lipopolysaccharide (LPS; Sigma-Aldrich, Castle Hill) and 20 μg/mL interferon γ (IFNγ; Sigma-Aldrich, Castle Hill) for 24 h.

### mRNA extraction from CD14^+^ PBMC, U-937, THP-1 and HL-60 cells

Messenger RNA was extracted from a cell pellet using the Isolate II RNA kit (Bioline, Alexandria) as per the manufacturer’s instructions. In brief, cells were lysed with 350 μL lysis buffer and 3.5 μL β-mercaptoethanol. Lysate was loaded onto an ISOLATE II filter and centrifuged at 11,000 x g in a 2 mL collection tube for 1 min. Then 350 μL of 70% ethanol was added to each collection tube, lysate loaded into an ISOLATE II Mini column with a 2 mL collection tube and centrifuged for 30 s at 11,000 x g. The flow through was discarded and 350 μL Membrane Desalting Buffer was added and centrifuged at 11,000 x g for 1 min. Reconstituted DNase 1 was added to Reaction Buffer at a 1:10 dilution. The solution was mixed and 95 μL was added to each column and incubated at room temperature (RT) for 15 min. The column was washed with 200 μL Wash Buffer 1 and centrifuged for 30 s at 11,000 x g. The Flow through discarded and the column washed twice with Wash buffer 2 with 600 μL for 30 s at 11 000 x g and then 250 μL for 2 min. The column was placed into a 1.5 mL collection tube and eluted with 30 μL RNase-free water by centrifugation at 11,000 x g for 1 min. The RNA yield was measured using a NanoDrop 1000 spectrophotometer (Thermo Fisher Scientific, Scoresby) and diluted to a final concentration of 166.6 ng/μL. All samples were frozen at -20°C until required.

### cDNA preparation and qPCR gene expression

Complementary DNA was prepared using the Tetro cDNA Synthesis Kit (Bioline, Alexandria), as per manufacturer’s instructions. In brief, the reaction mixture was prepared with 2 μL mRNA, 1 μL random hexamers primer, 1 μL dNTPs 10 mM, 4 μL 5 x RT buffer, 1 μL RNase inhibitor and 10 μL DECP treated water with 1 μL Tetro cDNA RT or 11 μL DECP treated water per sample. A negative RT control was used for each sample. PCR was performed on a Thermal Cycler (Biorad, Gladesville) on the following settings: 25°C for 10 min, 45°C for 30 min, 85°C for 5 min and stored at 4°C.

The qPCR reaction mixture was prepared using the SybrGreen I Master Mix (Roche, North Ryde) as per manufacturer’s instructions. In brief, 2 μL cDNA, 2 μL 5 μM forward and reverse primers, 5 μL SybrGreen 1 Master Mix (Roche, North Ryde) and 1 μL water per sample were added to a LightCycler 480 (Roche, North Ryde) 384-well plate. The qPCR was run using the Sybr Green protocol using the LightCycler 480 (Roche, North Ryde) using the following settings: 94°C for 2 min for 40 cycles, 94°C for 15 s, and 60°C for 1 min to measure primer melting temperature. All data were normalised against both housekeeper genes *GAPDH* and *ACTB2* individually before data were grouped, with a Ct value of >35 being deemed not detected. Primers (Gene Works, Melbourne) used for the study are described in **S1A Table**.

### CD surface marker expression and FACS analysis

Cells were re-suspended in assay buffer (PBS containing 1% bovine serum albumin; BSA) at a concentration of 250,000 cells in 200 μL. A volume of 200 μL of each primary mouse anti-human antibody (BD Biosciences, North Ryde) at a concentration of 1 μg/mL was incubated with the cells for 1 h at 4°C. Following this incubation cells were washed three times with assay buffer and re-suspended in 200 μL assay buffer containing 5 μg/mL of the secondary antibody (fluorescently tagged R-phycoerythrin (R-PE) conjugate Goat anti-Mouse IgG (H+L) secondary antibody; Life Technologies, Scoresby) and incubated for a further 1 h at 4°C. Following this incubation, the cells were washed three times with assay buffer and re-suspended in 500 μL assay buffer containing 5 nM Sytox Red (Thermo Fisher Scientific, Scoresby) which was used as a viability dye. Cells were analyzed using a FACS Canto II flow cytometer (BD Biosciences, North Ryde). PE was excited with by a blue laser (488nm) and detected by a 585/42 filter. FSC, SSC and APC voltages of 100, 400 and 269 were applied without any compensation. Antibodies (BD Biosciences, North Ryde) used for the study are described in **S1B Table**.

### Chemotaxis transwell assay

Chemotaxis assays were performed using HTS-transwell inserts (Sigma-Aldrich, Castle Hill). A volume of 150 μL of chemoattractant (monocyte chemoattractant protein-1; MCP-1, formyl-methionyl-leucyl-phenylalanine; fMLP, leukotriene B4; LTB-4, and monocyte inhibitory protein-1α; MIP-1α) in serum free growth medium was added to the bottom chamber of the insert. In the top chamber 50,000 cells re-suspended in 50 μL serum free growth medium were added. A negative control using vehicle and positive control using 10% FBS were included in each assay. Once the samples were prepared the plates were incubated to obtain an optimal window for either 3h for the CD14^+^ PBMCs or 4 h for the cell lines at 37°C with 5% CO_2_. Following the incubation, the transwells were removed and the plates dried before fixing of the cells with formalin solution that contained Hoechst 33258 (Sigma-Aldrich, Castle Hill) for nuclei staining. Wells were imaged using an InCell Analyser 2000 (GE Healthcare, Little Chalfont) and number of cells quantified using Image J (open source).

### Data analysis

Experimental data were analyzed using R version 3.4.1 (The R Foundation; differential gene expression), FlowJo V10 (LLC, Ashland, OR; FACS analysis), Prism 7.0a (GraphPad Software Inc., San Diego, CA; CD marker expression levels and chemotaxis) or Image J 1.50b (NIH; chemotaxis). All qPCR data were normalized against the housekeeper genes *GAPDH* and *ACTB2* as described by the equation below to produce ΔΔC_T_ values. The relative expression values were calculated compared to the relevant undifferentiated cell type.

$${Normalized\;Expression}_{sample(GOI)}=\frac{{RQ}_{sample(GOI)}}{{\left({RQ}_{sample\;(Ref\;1)}\times {RQ}_{sample(Ref\;2)}\right)}^{\frac{1}{n}}}$$

Where RQ is the relative quantity of a sample, Ref is the reference target in that experiment that includes a reference target in each sample; where Ref 1 is *GAPDH* and Ref 2 is *ACTB2*, and GOI is the gene of interest and ‘n’ is the number of reference targets.

**Fig 1:**
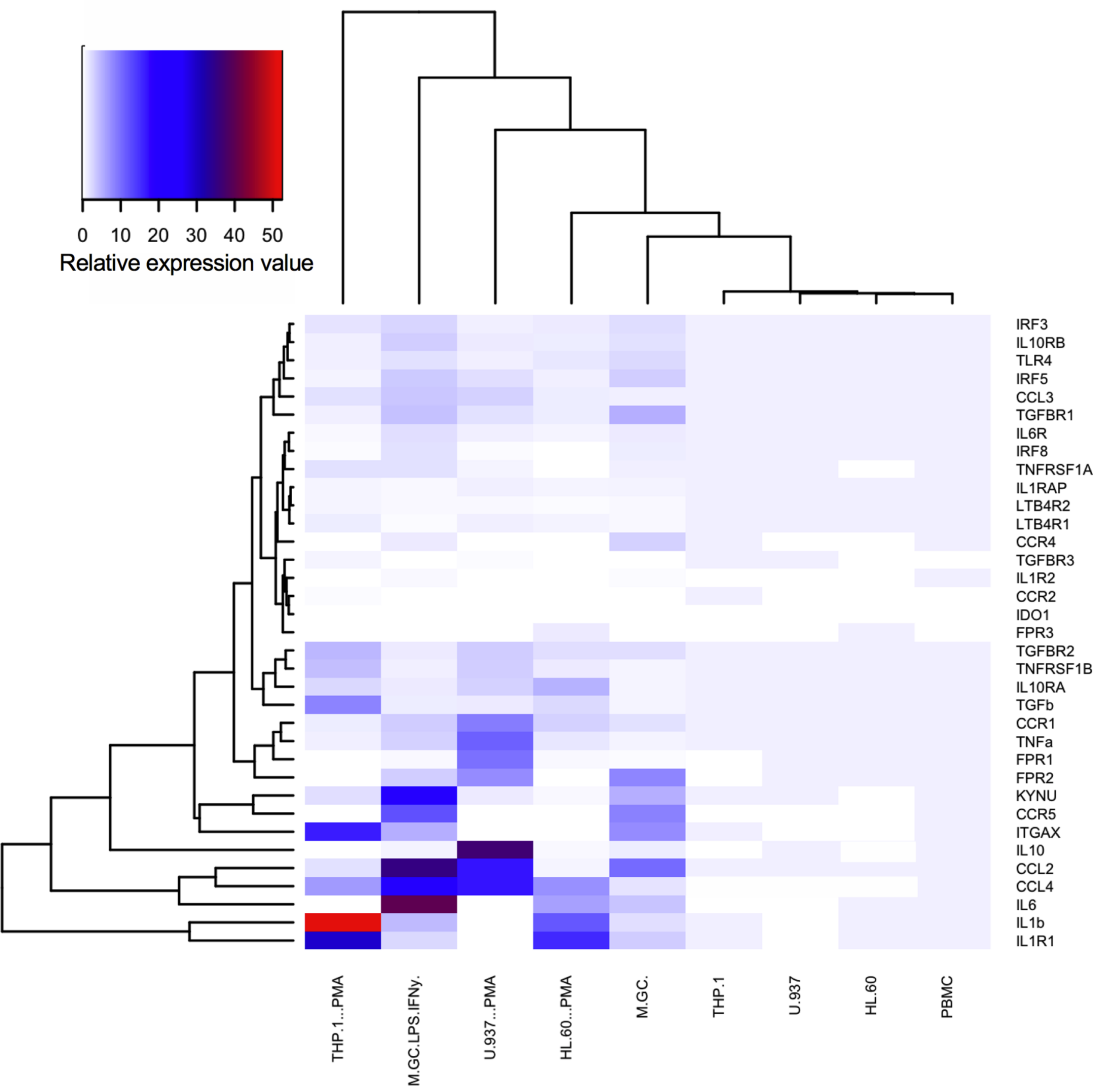
Hierarchical clustered heat map of mean relative expression values for undifferentiated and differentiated CD14^+^ human peripheral blood mononuclear cells (PBMC, M(GC) and M(GC)LPS/IFNγ) and monocyte-like cell lines (MCLCs ± PMA) including THP-1, U-937 and HL-60s. PBMCs were differentiated using 10 ng/mL granulocyte-macrophage colony stimulating factor (GM-CSF) for 6 days to give M(GC) and activated using 100 ng/mL LPS and 20 μg/mL IFNγ for 24 h to generate M(GC)LPS/IFNγ. MCLCs were differentiated using 16 ng/mL phorbol-12-myristate-13-acetate (PMA) for 48 h. Data represent n = 3–10; raw data, including the relative expression ± SEM used to generate this heat map, are shown in **S2 and S3 Tables**. All qPCR data were normalized against both housekeeper genes *GAPDH* and *ACTB2* and the values calculated as described in the Methods. Relative expression values were then calculated versus the relevant non-differentiated cell type. Where no expression was detected a value of 0 was used. Thirty-five genes that encode for inflammatory chemokines, cytokines, adipokines and their relevant receptors were selected as they are associated with inflammation or have been implicated in the development and/or progression of obesity-induced insulin resistance. In addition, small subsets of genes encoding for regulatory factors and enzymatic processes that have been implicated in the pathogenesis of T2DM were profiled. Differential gene expression data were analysed using R version 3.4.1 and hierarchical clustering was performed using complete linkage method with Euclidean distance measure.

Where no expression was detected a value of 0 was used. Data are shown graphically as the mean with hierarchical clustering performed using complete linkage method with Euclidean distance measure. Mean raw Ct values ± SEM values are reported in **S2 Table** and the mean relative expression values ± SEM used to generate the heat map in **Fig 1** is reported in **S3 Table**. Flow cytometry data was gated against a negative control and the CD surface marker expression data expressed as %PE positive cells and as the mean level of PE emission; both were expressed as mean ± SEM (n = 3–5). Data for the chemotaxis transwell migration assays were calculated as the chemotactic index (sample wells / basal wells from the same cell line i.e. each cell line’s response was normalised to its own baseline) and represented as the mean ± SEM (n = 3–22).

#### Statistical and principal component analysis

Changes in the gene expression values analysed by one-way ANOVA with Tukey post-hoc multiple comparison test compared to basal, with *P<0.05, **P<0.01, ***P<0.001 and ****P<0.0001 deemed significant. Principal component analysis (PCA) was subsequently applied to the calculated the relative expression values, using singular value decomposition as implemented in the package scikit-learn [22]. This analysis is a dimensionality reduction method that uses transformations to project a high-dimensional set of data into a lower dimensional set of variables, called principal components (PCs) [23]. The extracted PC values contain important information from the data, revealing its internal structure in a way that best explains its variance [24]. PCs are ranked according to the percentage of total variance in the data they explain. PC1 explains the maximal total variance found within the data. The subsequent PC values represent the remaining variation, without being correlated with the preceding components. The script used for the analysis and plotting can be found at https://github.com/thomas-coudrat/pca_analysis.

#### Compounds and reagents

The chemoattractants were purchased from Sigma-Aldrich (Castle Hill, NSW), Sapphire Biosciences (Redfern) and In Vitro Technologies Pty Ltd (Noble Park). Primer probe sets were purchased from Geneworks (Melbourne). Cell culture and molecular biology reagents were supplied by Thermo Fisher Scientific (Scoresby).

#### Nomenclature

In the body of the text proinflammatory macrophages relate to monocytes differentiated with GM-CSF. However, in keeping with the suggested modification of the nomenclature the figures use PBMC, M(GC) and M(GC)LPS/IFNγ [3]. For the MCLCs, ‘+ PMA’ represents the differentiated cell type.

## Results

### Hierarchical clustering of gene expression data

Thirty-five genes that encode for inflammatory chemokines, cytokines, adipokines and their cognate receptors were selected for analysis (**S1A Table**). These were chosen based on their association with inflammation or involvement in the development and/or progression of obesity-induced insulin resistance. In addition, small subsets of genes encoding for regulatory factors and enzymatic processes that have been implicated in the pathogenesis of type 2 diabetes mellitus (T2DM) were profiled, including interferon regulatory factors (*IRF*), indoleamine 2,3-dioxygenase (*IDO1*) and kynureninase (*KYNU*). Raw gene expression data were normalized to the housekeeping genes, *GAPDH* and *ACTB2* (as described in the Methods) to estimate relative expression values, which were subjected to hierarchical analysis. A comparison of the relative expression values using heat map analysis revealed a clear segregation of absent (white), low (blue) and high (red) expressing genes across all cell types (**Fig 1**). Data used to generate this heat map can be found in **S3 Table**. There was a clear subset of genes that appear to be highly expressed in CD14^+^ peripheral blood mononuclear cells (PBMCs) but which are absent in the THP-1, HL-60 and U937 monocyte-like cell lines (bottom right quadrant; **Fig 1**). Surprisingly, these include *IL6* and *IL10*, two of the most highly studied, and relevant chemokines involved in inflammation. In addition, genes associated with enzymatic processing of tryptophan, *KYNU* and *IDO1*, were also both absent. Clustering analysis of the different cell types revealed, as expected, significant differences between the undifferentiated and differentiated cells types.

**Fig 2:**
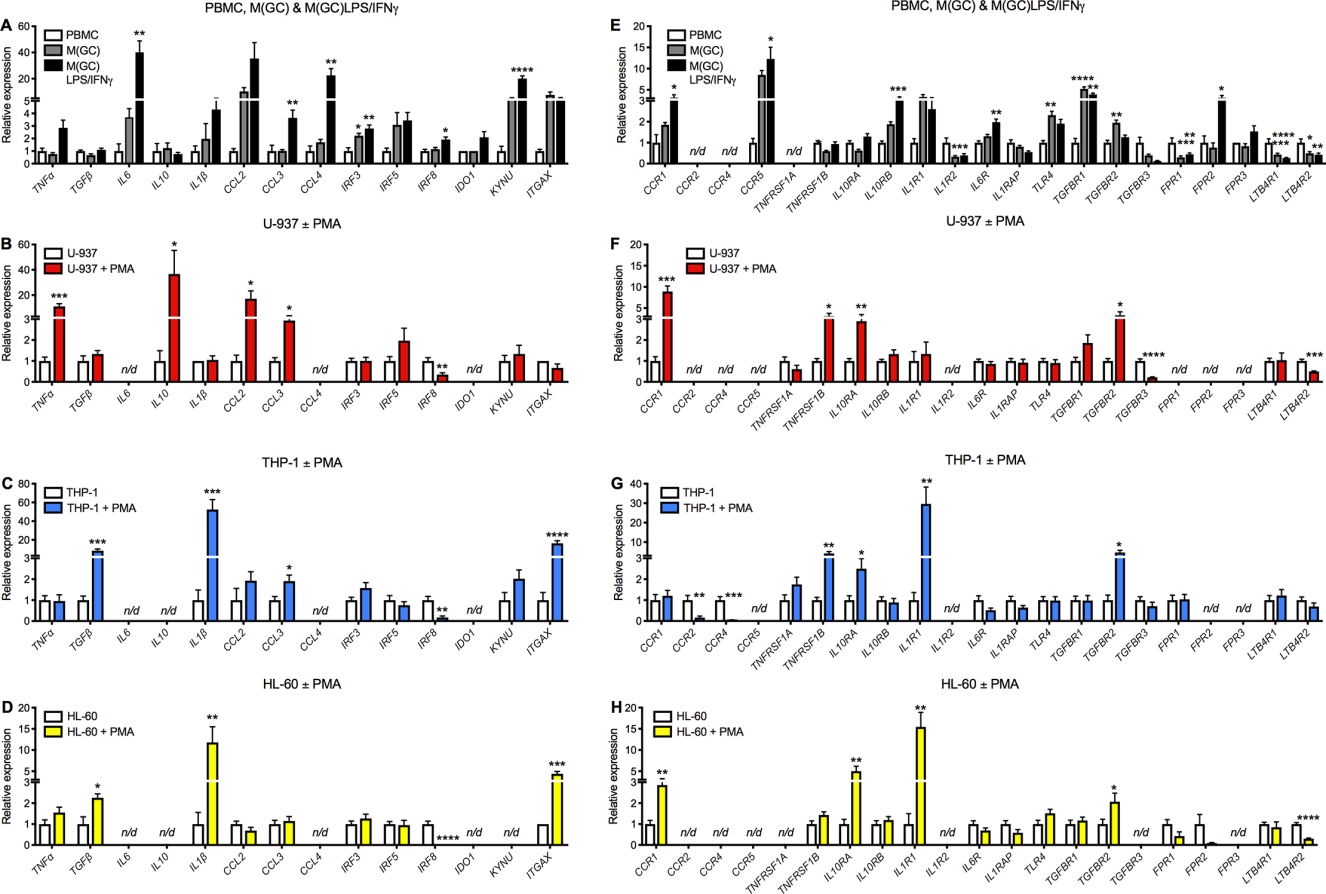
Relative expression analysis of chemokines, cytokines, receptors, enzymes, and regulatory factors for undifferentiated, differentiated and polarized human peripheral blood monocytes (PBMCs; A and E; white, grey and black bars respectively), and monocyte-like cell lines (MCLCs) including U-937 (B and F; red bars), THP-1 (C and G; blue bars), and HL-60 (D and H; yellow bars). PBMCs were differentiated using 10 ng/mL granulocyte-macrophage colony stimulating factor (GM-CSF) for 6 days to give M(GC) and activated using 100 ng/mL LPS and 20 μg/mL IFNγ for 24 h to generate M(GC)LPS/IFNγ. MCLCs were differentiated using 16 ng/mL phorbol 12-myristate 13-acetate (PMA) for 48 h. Grouped data are shown as mean ± SEM (n = 3–10). Relative expression data including the mean ± SEM are shown in **S3 Table**. Genes not detected were marked as *n/d*. Statistical significance was determined by one-way ANOVA with Tukey’s multiple comparison test compared to undifferentiated cells for the PBMCs, or by Student’s t-test for the MCLCs, with *P<0.05, **P<0.01, ***P<0.001 and ****P<0.0001 deemed significant.

To illustrate these findings more clearly, each cell type has been grouped along with its respective differentiated state, and changes in gene expression expressed as relative expression values (**Fig 2A, 2B, 2C, 2D, 2E, 2F, 2G and 2H;** *n/d* denotes ‘not detected’). Data used to generate these figures can be found in **S3 Table**. In the PBMCs there is a clear increase in *IL6*, *CCL3*, *CCL4*, *KYNU* and several of the cytokine receptors (**Fig 2E**). The MCLCs show a very different profile compared to the PBMCs and each other with respect to chemoattractant gene expression; in the U-937s *TNFα*, *CCL2* and *CCL3* are significantly upregulated, whilst in both the THP-1 and HL-60s both *TGFβ* and *IL-1β* are significantly increased. A more similar expression profile is observed for the chemokine receptors across all three MCLCs. Interestingly, a significant downregulation of the interferon regulatory factor 8 (*IRF8*) was observed in all MCLCs. This gene is involved in monocyte lineage and typically is downregulated upon monocyte differentiation into a macrophage. Collectively these demonstrate that upon PMA treatment MCLCs differentiate into a macrophage-like cell type.

**Fig 3:**
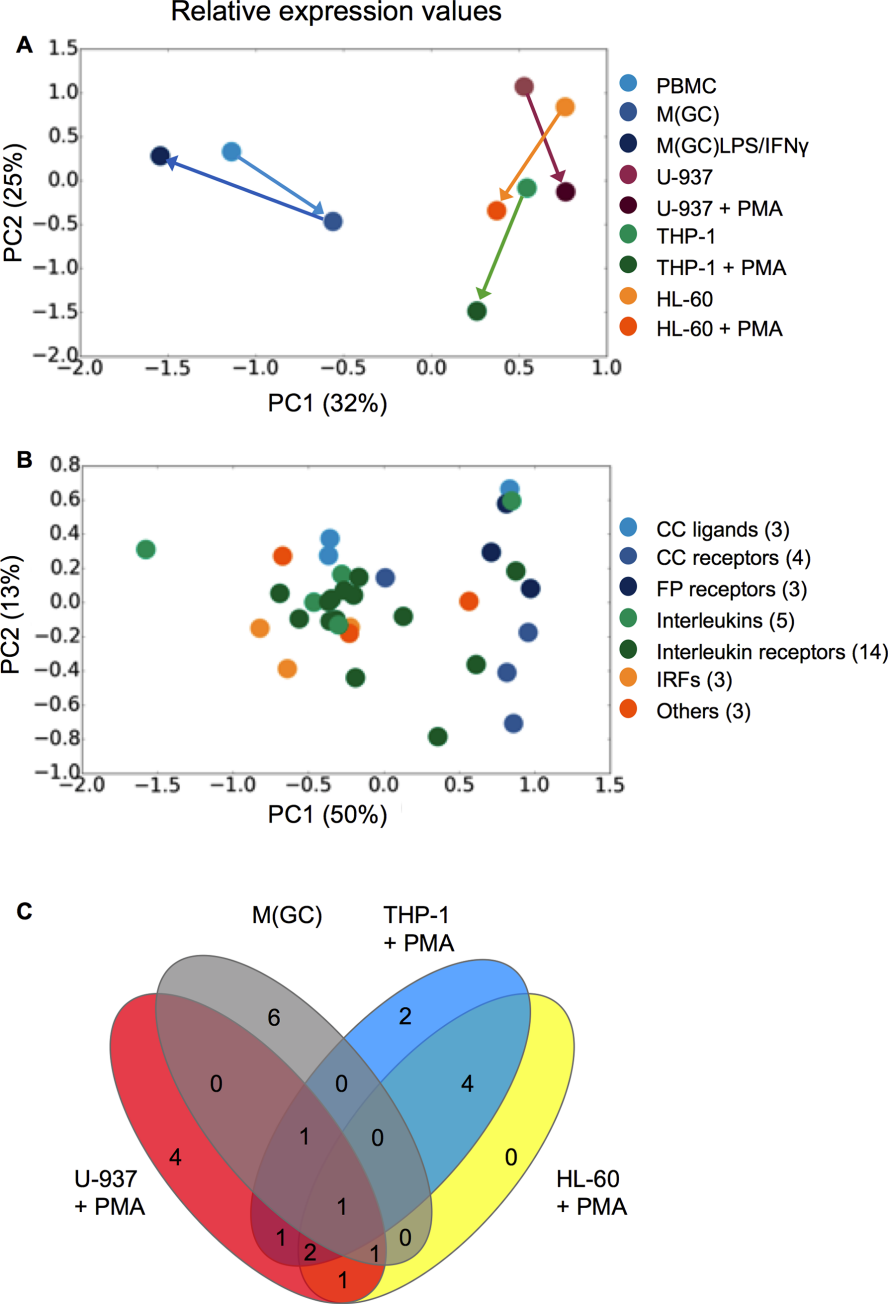
Principal component analysis (PCA) of the mean relative expression values (n = 3–10) grouped by either cell type (A) or gene family (B) shown in Fig 1 and (C) Venn diagram using the relative expression values of genes displaying a significant up-regulation or down-regulation in expression, after GM-CSF or PMA treatment for PBMCs, U-937, THP-1, and HL-60 cells. Connecting arrows in the PCA analysis represent changes from undifferentiated to differentiated state. Data used to generate (**C**) are summarized in **Table 1**.

To analyse these data sets in a non-biased approach a principal component analysis (PCA) was performed. PCA is a dimensionality reduction method that reveals values in the dataset that contribute variability between the combined data sets including both undifferentiated and differentiated cells, and individual genes. Cell lines and / or genes that display similar behaviour cluster together. This analysis revealed that human PBMCs cluster separately from all immortalised cells for the relative expression values (blue symbols and arrows; **Fig 3A**), indicating that the human primary cells are genotypically distinct from all of the monocyte-like cell lines. However, as expected, a second clustering was evident between the non-PMA-treated and PMA-treated cells (burgundy, green and orange symbols and arrows), again correlating with those data obtained from the hierarchical clustering analysis. The composite of both PC1 (67%) and PC2 (12%) accounted for 79% of the total variability, indicating limited variation within the Ct dataset.

When PCA was performed to examine clustering of gene families across all differentiated cells (irrespective of cell type), no clustering was seen at the level of the calculated relative expression values (**Fig 3B**). Although in the latter PC analysis there does appear to be two “clusters; PC1–1.0–0.0 and 0.5–1.0”, they bear no relationship to the functional role(s) of the genes. These representations broadly represent what is seen in **Fig 1**; namely that the human PBMCs (whether differentiated or polarised) cluster separately from the monocyte-like cell lines, again irrespective of their treatment.

In order to further interrogate the data in **Fig 2,** genes which showed a statistically significant up or down-regulation following GM-CSF or PMA-treatment (compared to their undifferentiated state) were represented graphically as a Venn diagram (**Fig 3C**) and summarised in **Table 1**. This allows for comparison of genes with common and significant changes in expression between undifferentiated and differentiated cells across different cell backgrounds. As **Table 1** shows, the number of differentially expressed genes appears to be reasonably conserved across the cell backgrounds upon differentiation (between 9 and 16 genes). However, as **Fig 3C** reveals, there is limited overlap in the genes that are regulated across cell backgrounds. *TGFBR2* is regulated in a similar manner across the GM-CSF-treated PBMCs and PMA-treated MCLCs, whilst *LTB4R2* is downregulated in all cell types except PMA-treated THP-1s. Overall there is greater overlap amongst the immortalised MCLCs, with *IRF8* and *IL10RA* being similarly regulated (in addition to *TGFBR2*), whilst *TGFβ*, *IL1β*, *CCL3*, *ITGAX*, *TNFRSF1B* and *IL1R1* are similarly regulated in in at least two of the three MCLCs. These data further confirm that whilst the MCLCs are similar to each other, they are genotypically distinct from human primary monocytes and macrophages.

**Table 1:** Significant upregulation or downregulation in the relative gene expression levels compared to the un-differentiated cell of monocyte-like cells lines and human macrophages upon activation by either 20 nM PMA for 24 h for the cell lines, and 10 ng/mL GM-CSF for 6 days with or without polarization using 10 ng/mL LPS and 20 ng/mL IFNγ. Summary data used to generate the Venn diagrams as shown in **Fig 3**.

Class	Gene	M(GC) (n = 9–10)	U-937 + PMA (n = 3)	THP-1 + PMA (n = 3)	HL-60 + PMA (n = 3)
**Chemokines & cytokines**	*TNFα*	*n/s*	↑	*n/s*	*n/s*
	*TGFβ*	*n/s*	*n/s*	↑	↑
	*IL6*	*n/s*	*n/d*	*n/d*	*n/d*
	*IL10*	*n/s*	↑	*n/d*	*n/d*
	*IL1β*	*n/s*	*n/s*	↑	↑
	*CCL2*	*n/s*	↑	*n/s*	*n/s*
	*CCL3*	*n/s*	↑	↑	*n/s*
	*CCL4*	*n/s*	*n/d*	*n/d*	*n/d*
**Regulatory factors**	*IRF3*	↑	*n/s*	*n/s*	*n/s*
	*IRF5*	*n/s*	*n/s*	*n/s*	*n/s*
	*IRF8*	*n/s*	↓	↓	↓
**Enzymes**	*IDO1*	*n/s*	*n/d*	*n/d*	*n/d*
	*KYNU*	*n/s*	*n/s*	*n/s*	*n/d*
	*ITGAX*	*n/s*	*n/s*	↑	↑
**Receptors**	*CCR1*	*n/s*	↑	*n/s*	↑
	*CCR2*	*n/d*	*n/d*	↓	*n/d*
	*CCR4*	*n/d*	*n/d*	↓	*n/d*
	*CCR5*	*n/s*	*n/d*	*n/d*	*n/d*
	*TNFRSF1A*	*n/d*	*n/s*	*n/s*	*n/d*
	*TNFRSF1B*	↓	↑	↑	*n/s*
	*IL10RA*	*n/s*	↑	↑	↑
	*IL10RB*	*n/s*	*n/s*	*n/s*	*n/s*
	*IL1R1*	*n/s*	*n/s*	↑	↑
	*IL1R2*	↓	*n/d*	*n/d*	*n/d*
	*IL6R*	*n/s*	*n/s*	*n/s*	*n/s*
	*ILRAP*	*n/s*	*n/s*	*n/s*	*n/s*
	*TLR4*	↑	*n/s*	*n/s*	*n/s*
	*TGFBR1*	↑	*n/s*	*n/s*	*n/s*
	*TGFBR2*	↑	↑	↑	↑
	*TGFBR3*	*n/s*	↓	*n/s*	*n/d*
	*FPR1*	↓	*n/d*	*n/s*	*n/s*
	*FPR2*	*n/s*	*n/d*	*n/d*	*n/s*
	*FPR3*	*n/s*	*n/d*	*n/d*	*n/d*
	*LTB4R1*	↓	*n/s*	*n/s*	*n/s*
	*LTB4R2*	↓	↓	*n/s*	↓

n/d = not detected, n/s = not significant

In most cases the direction of regulation of genes across the different cell types is conserved, however, there are some notable exceptions. For example, *FPR1* is upregulated in PMA-treated U-937 cells, whilst it is downregulated in the GM-CSF-differentiated proinflammatory macrophages; *CCR2* and *CCR4* are downregulated in PMA-treated THP-1 cells and upregulated in the GM-CSF-differentiated proinflammatory macrophages. Deming linear correlation of the Ct or relative expression values between each of the different cell types, or between the undifferentiated and differentiated cell types, did not reveal any significant correlations (*data not shown*) indicating a wide range of diversity in the expression profiles of these cell types. To understand whether changes in mRNA levels between the cell backgrounds translated in to changes in phenotype, a panel of cluster of differentiation (CD) markers was investigated. As the MCLCs had similar gene expression profiles and THP-1 and U-937 cell lines are most widely used in the literature, only these two immortalised cell lines were used for CD marker expression and functional studies.

### Changes in CD surface marker expression by FACS analysis

**Fig 4:**
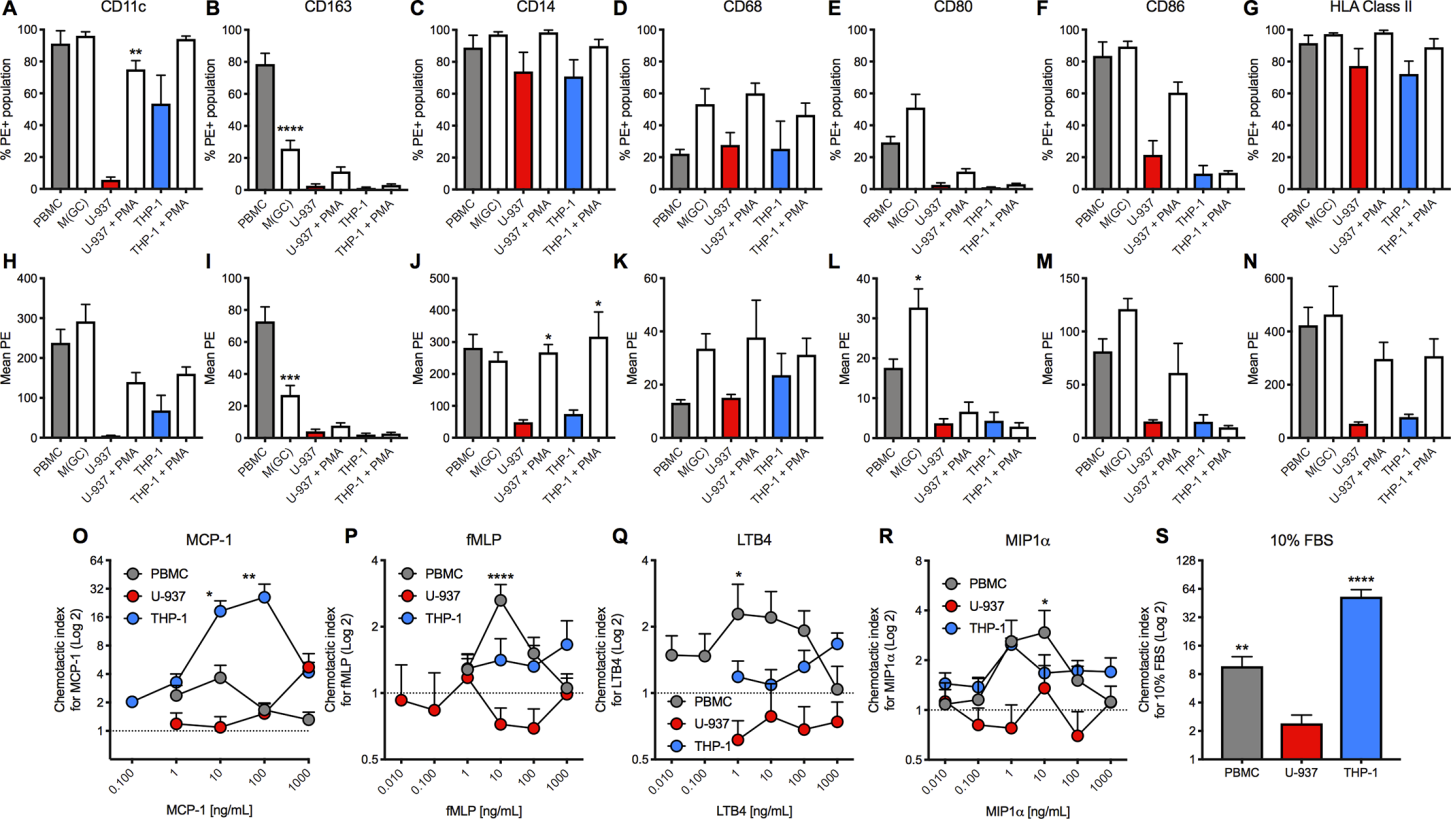
Effects of differentiation treatment on cluster of differentiation (CD) markers expression on human peripheral blood monocytes (PBMCs; grey bars) and monocyte-like cell lines (MCLCs; U-937; red bars and THP-1; blue bars) and concentration-response curves to proinflammatory chemoattractants using a transwell migration assay using PBMCs (grey circles), U-937 (red circles) and THP-1 (blue circles) cells. Data are expressed as % cells expressing (%PE+ population) for (**A**) CD11c, (**B**) CD163, (**C**) CD14, (**D**) CD68, (**E**) CD80, (**F**) CD86 and (**G**) HLA Class II, or as the relative level of expression (mean PE) for (**H**) CD11c, (**I**) CD163, (**J**) CD14, (**K**) CD68, (**L**) CD80, (**M**) CD86 and (**N**) HLA Class II. Grouped data are shown as mean ± SEM (n = 3–5). Data are expressed on a Log2 scale as the chemotactic index compared to basal levels following 3–4 h treatment with the various chemoattractants using (**O**) MCP-1, (**P**) fMLP, (**Q**) LTB4, (**R**) MIP1α and (**S**) 10% FBS. Grouped data are shown ± SEM (n = 3–22). Statistical significance was determined by one-way ANOVA with Tukey’s multiple comparisons test compared to undifferentiated cells for the CD markers, and two-way ANOVA with Dunnett’s multiple comparison tests compared to vehicle control for chemotaxis, with *P<0.05, **P<0.01, ***P<0.001 and ****P<0.0001 deemed significant.

To enable a comparison of the CD marker expression profile across monocytic-like cell lines and human PBMCs (both before and after differentiation), seven CD surface markers were selected for FACS analysis, including those that distinguish between the classical proinflammatory (CD68^+^/CD163^-^) and alternative anti-inflammatory (CD68^+^/CD163^+^) macrophage phenotypes. Levels of expression are represented as either the percentage of the total cell population expressing the marker of interest (%PE+ population; **Fig 4A, 4B, 4C, 4D, 4E, 4F and 4G**) or as a relative level of expression (mean PE; **Fig 4H, 4I, 4J, 4K, 4L, 4M and 4N**) for both undifferentiated and differentiated cells, as for the gene expression analysis.

There was more concordance in the expression of CD markers for THP-1 and U-937 cells compared to PBMCs, though absolute expression levels varied considerably. This was best highlighted by the changes induced under differentiating conditions; there are limited increases in the levels of CD marker expression in the GM-CSF-differentiated proinflammatory macrophages compared to PBMCs, presumably due to high basal expression levels for most of the markers in the original monocytic state. As expected (from a CD14^+^ enriched population), the levels of CD14 and CD11c expression in the PBMCs were high. Differentiation of PBMCs significantly decreased CD163, a haptoglobin-hemoglobin (Hp-Hb) scavenger receptor, and increased CD68 and CD80 cell surface expression. These changes for CD163, CD80, and the pan marker CD68 levels confirm differentiation of these cells in to a pro-inflammatory macrophage/DC-like phenotype.

Data from THP-1 and U-937 cell lines appears to be similar for both the %PE+ population and the total levels of expression of CD markers. These cell lines also display similar changes in CD marker expression profile between the un-differentiated and differentiated state, although the changes tend to be more marked for the U-937 cells (**Fig 4**). There is an increase in CD11c in both cell lines, albeit not significant in the THP-1 cells due to their high basal expression level. *ITGAX* encodes CD11c (integrin subunit alpha x) and was upregulated in the gene expression analysis, consistent with the CD surface expression data. HLA (MHC class II) expression, which is found on antigen presenting cells including DC, B cells and mature macrophages, was also increased upon differentiation of both THP-1 and U-937 cells. There is no change in the expression of this marker in the differentiated PBMCs, reflecting the high basal levels found in the undifferentiated state (**Fig 4N**). More generically this is a trend that separates the monocyte-like cell lines and the PBMCs, whereby the levels of the CD surface markers appear to much higher for the latter, including CD11c, CD163 and HLA (MHC Class II). Due to this high level of basal expression there is no significant change upon GM-CSF treatment.

However, upon differentiation (with PMA for the monocyte-like cell lines and GM-CSF treatment for the PBMCs), the pattern of CD marker expression is more similar between the PBMC and U-937s, with CD11c, CD14, CD68, CD86 and HLA all expressed at moderate levels. THP-1 cells show a similar expression profile except the DC markers, CD80 and CD86 do not appear to be changed under differentiation conditions. These data imply that the U-937 cell line, and to a lesser degree THP-1s, may also contain a mixed proinflammatory macrophage/DC-like phenotype. Although the correlation is not exact (for example, CD86 expression is low in THP-1 cells and CD163 is different for PBMCs versus THP-1 and U-937 cells), the overall pattern suggests that at the level of CD marker expression, the monocyte-like cell lines more faithfully replicate the human PBMC profile than at the level of inflammation-relevant gene expression.

### Chemotactic responses

One of the major functions of monocytes is their ability to migrate to sites of inflammation, injury and infection in response to the release of various inflammatory mediators including chemokines and adipokines; this is one of the central tenets underlying obesity-induced inflammation and insulin resistance. To investigate how these gene and CD marker similarities and differences were reflected in chemotaxis, the cells’ ability to migrate across a chemoattractant gradient was studied using a transwell assay system. In the above studies both undifferentiated and differentiated cells were used, however, it is well known that PMA treatment of U-937 and THP-1 cells causes rapid adhesion to plastic surfaces [25], so only non-PMA treated cells were used for chemotaxis. Likewise, GM-CSF differentiated macrophages also adhere rapidly and become largely immobile and so only undifferentiated PBMCs were studied. Concentration-response curves to a panel of chemoattractants (monocyte chemoattractant protein-1; MCP-1, formyl-methionyl-leucyl-phenylalanine; fMLP, leukotriene B4; LTB-4 and monocyte inhibitory protein-1α; MIP-1α; **Fig 4O, 4P, 4Q and 4R**) were generated, using the migratory response to 10% foetal bovine serum (FBS) as a positive control (**Fig 4S**). Data were expressed as the chemotactic index, whereby the basal response in the same cell line in the absence of chemoattractant is equal to 1.

PBMCs responded equally to all chemoattractants with a characteristic bell-shaped curve [26] and maximal effect at 10 ng/mL for all treatments (**Fig 4O, 4P, 4Q and 4R**). U-937 cells appeared functionally impaired with little or no migration in response to the same panel of chemoattractants (**Fig 4O, 4P, 4Q and 4R**), with only a moderate response observed at high concentrations of MCP-1 (1000 ng/mL; **Fig 4O**). These cells also failed to respond significantly to the positive control, FBS (**Fig 4S**). The THP-1 cells however, exhibited a marked MCP-1 response with a peak chemotactic index of 26.0 ± 9.8 at 100 ng/mL, although a strong response was also evident at 10 ng/ml (18.5 ± 4.9; **Fig 4O**). A small yet significant effect was also observed for MIP-1α (**Fig 4R**), which is in line with expression of their requisite target receptors, *CCR1*, *CCR4* and *CCR5* (**Fig 2E, 2F, 2G and 2H**).

## Discussion

The human-derived MCLCs, U-937, THP-1 and HL-60, are routinely used as surrogates of monocytes isolated from human peripheral blood mononuclear cells (PBMCs) [27]. These cell lines have been variously characterised based on the mRNA expression levels of inflammatory mediators, ion channels and G protein-coupled receptors (GPCRs) [16, 18]. However, in recent years many of these cell lines have been used to study the role of immune cells in metabolic diseases, including type 2 diabetes and obesity [19]. To systematically evaluate MCLCs as cellular models of human primary monocytes and macrophages, we compared inflammation-relevant mRNA levels, cell surface CD marker expression and chemotactic responses to major chemokines. Gene expression analysis suggested that most genes were present at similar levels (Ct and relative expression values) across all undifferentiated cells, data that were confirmed using a principal components analysis. However, key genes *KYNU*, *IL6*, *IL10*, *CCL4* and *IL1R2* were either not expressed or found at very low levels in monocyte-like cell lines, but found in PBMCs, suggesting that at the mRNA expression level there are some crucial differences between the immortalised and primary cells. Clearly this limited number of genes analysed would only capture a small proportion of changes observed during monocyte differentiation. However, the genes chosen selectively encapsulate a range of interleukins, chemokines and cytokines (ligands and their cognate receptors) as well as regulatory factors and enzymes implicated in the pathogenesis of T2DM. A follow-up study performing whole genome analysis using RNAseq would enable a more comprehensive overview of the gene changes involved in monocyte differentiation. For example, IL-8 has been widely reported to be upregulated in T2DM patients who demonstrate both a worse inflammatory and cardiometabolic profile [28]. In addition, IL-8 has been shown to be upregulated in PMA-treated U-937 cells [29], clearly indicating a potential role for this chemokine in immuno-metabolism. The pattern of CD marker expression was more similar for THP-1 and U-937 cells compared to PBMCs, especially after differentiation, though absolute expression levels varied considerably. In functional transwell assays, PBMCs and THP-1s both migrated in response to a panel chemoattractants, though the former migrated equally with MIP-1α, LTB-4, fMLP and MCP-1, whereas the THP-1 cells responded only to MCP-1 and MIP-1α. However, despite a similar gene and CD expression profile to THP-1 cells, U-937 cells were functionally impaired, with minimal/no migration in response to any chemoattractant. The HL-60 cell line was not evaluated beyond differential gene expression analysis as it yielded only moderate changes in its mRNA expression profile upon PMA treatment and it is less routinely used to model monocyte biology compared to THP-1 and U-937 cells. Furthermore, the HL-60 cell line was derived from an earlier progenitor stage (promyelocytes) and this may explain the reduced number of changes in gene expression observed following PMA treatment. This is perhaps unsurprising as issues surrounding HL-60 cells have been described previously, including a lack of superoxide generation, bacterial ingestion, and complement secretion when treated with PMA [30], indicating that these cells are perhaps not suitable for investigation into monocyte / macrophage biology.

This study is the first head-to-head comparison incorporating both a genotypic and phenotypic characterisation of PBMCs and multiple monocyte-like cell lines. Thus, it avoids the pitfalls of inter-laboratory comparisons of multiple cell types and analyses in the published literature. Our findings show clear similarities and differences to previous studies. PBMCs express high CD14^+^ levels (due to the enrichment isolation method used) and PMA increases CD14^+^ expression in both THP-1 and U-937, which is in agreement with some previous studies in both cell lines [25, 30]. However, these findings are in stark contrast to Baek and colleagues [31], who showed no up-regulation of CD14^+^ in U-937 cells, rather only in M-CSF-differentiated macrophages. Why these differences are observed is unclear but is most likely due to variations in tissue culture conditions, growth medium constituents, differentiation protocols and treatment times, illustrating the difficulties in comparing published data sets with such cell lines. A universal differentiation protocol could be implemented for these cell lines, similar to those already proposed by a consortium of macrophage biologists, who published a nomenclature and experimental guidelines suggesting ways to overcome such issues [3]. Although this study focuses on primary monocytes and macrophages, perhaps a similar approach could be applied to these commonly used monocyte-like cell lines, especially as other differentiation reagents, including DMSO and VitD3, have been used [18]. Clearly this study raises some key issues when comparing GM-CSF-differentiated PBMCs and PMA-treated MCLCs; these stimuli clearly will activate very different pathways. Nonetheless, PMA-treated monocyte like cell lines are still often used as surrogates for GM-CSF-stimulated PBMCs (as both of these treatments generate a ‘pro-inflammatory macrophage’ phenotype). Herein it was our intention to determine to what degree PMA-treated monocyte cell lines reflect the phenotype of GM-CSF-differentiated PBMCs in the context of metabolic inflammation. It would be theoretically desirable to comprehensively extend the study to examine the reciprocal treatments (i.e. PMA-treated PBMCs and GM-CSF-treated monocyte cell lines). However, unfortunately, PMA treatment of PBMCs causes a downregulation of both CD4 and CD14 expression and has limited effects on cytokine production [32]. Moreover, the expression of the GM-CSF receptor CD116 appears to be low in the MCLCs (including THP-1 cells), rendering it unlikely to have any significant effect.

As these cell lines are used as surrogates to PBMCs to interrogate inflammation pathways, especially many of those shown to be involved in immuno-metabolism, the receptors to some key chemokines were also included in this study. Down regulation of various GPCRs, including *CCR2* and *CCR5*, has been shown when a Ficoll gradient is used during monocyte isolation [33, 34]. Consistent with this, we observed limited expression of these receptors in our isolated PBMCs. The reduced expression may explain the moderate chemotactic response observed to MCP-1 in the PBMCs in comparison to THP-1 cells. A similar loss of *CCR6 and CXCR3* expression in isolated monocytes from healthy donors using Ficoll-gradient isolation has also been reported [34] and our data supports these findings. It remains unknown whether other genes expressed in the PBMCs could be affected by the isolation process, notably *TGFBR3* and *FPR1*; genes that encode for the receptors of the key pro-inflammatory mediators TGFβ and fMLP, respectively.

In addition to chemokines and cytokines, various enzymes have been implicated in the pathophysiology of immune regulation [35], including kynureninase (*KYNU*; which is elevated in serum of diabetic patients [36]) and indoleamine 2,3-dioxygenase (*IDO1*, which along with *KYNU* regulates the catabolism of tryptophan). In our study *KYNU* and *IDO1* were expressed at low levels in PBMCs but were upregulated in the GM-CSF-differentiated proinflammatory macrophages and the LPS/IFNγ-activated macrophages. In the MCLCs *KYNU* was expressed at low levels, which did not significantly change following PMA treatment. However, *IDO1* was not expressed in any immortalised cell line even following PMA treatment. As IDO1 is involved in tryptophan metabolism and production of kynureninase [37], its absence suggests that the immortalised cell lines may not be suitable for the study of these immunoregulatory pathways.

Analysis of CD surface marker expression indicated that the PBMCs and monocyte-like cell lines display a similar phenotype, albeit with higher basal levels of predominant markers (CD11c^+^, CD14^+^ and HLA^+^) [38] on PBMCs compared to THP-1 and U-937 cells. As expected, GM-CSF-differentiated macrophages showed a significant increase in the expression of CD68 and CD80, two classical macrophage/DC markers [39], and a decrease in the anti-inflammatory scavenger receptor, CD163 [40]. With PMA treatment, a clear increase in the levels of CD11c, CD14 and HLA, similar to that found on antigen presenting cells such as DC, B cells and macrophages, was observed in THP-1 and U-937 cells. These changes also correlated with changes in gene expression. For example, *ITGAX*, the gene for CD11c, was expressed at high levels, and further increased under differentiating conditions. These findings suggest that with regard to the CD markers, undifferentiated THP-1 and U-937 cells poorly replicate the profile of PBMCs, but that post-differentiation these differences are reduced, suggesting that PMA-treated monocyte-like cell lines more closely resemble a GM-CSF differentiated proinflammatory macrophage [18].

Based on the gene and CD surface expression, THP-1 and U-937 cells are similar to one another, but different to CD14^+^ PBMCs. However, in the functional chemotaxis assay a very different profile was observed. Both the THP-1 cells and PBMCs produced prototypical bell-shaped response curves to a panel of proinflammatory chemoattractants, similar to published data [41, 42]. Surprisingly, the U-937 cells failed to respond to all the chemoattractants tested, including 10% FBS. Kew and colleagues [43] demonstrated that these cells did not respond to the chemoattractant agents fNLP, IL-8 and LTB4, but did migrate when their cognate receptors, Ca5R or fNLP, were over-expressed. Similarly, Ott and colleagues [44] demonstrated migration to CXCL12, a molecule that is known to be involved in monocyte differentiation and migration [45], but again only when its receptor, CXCR4, was over-expressed. Therefore, an explanation for this functional impairment may be low expression levels of the relevant receptors. Gene expression data shows that Ct values of the receptors for each of the four chemoattractants used in this study ranged from 25 to >35. However, whilst a direct measure of chemokine receptor expression is not available at the level of protein, the mRNA expression levels in the U-937 cells are similar to those in both the THP-1 and PBMCs that responded to chemoattractants. These data suggest that there is a disparity between mRNA for chemoattractant receptors and their subsequent function, implying other levels of regulation of protein expression. The absence of response even to FBS, which contains a wide range of chemoattractants, also supports these findings and indicates that these cells may not be suitable for functional migration studies using inflammatory mediators. It should be noted that migration assays are just one example of a functional response; to compliment these findings alternative functional assessment could be performed, including cytokine release, cell proliferation or downstream second messenger signalling assays.

## Conclusion

Collectively the gene expression data, CD marker profile and functional chemotaxis assays suggest that the monocyte-like cell lines, THP-1 and U-937, only partly replicate freshly isolated human CD14^+^ PBMCs in their undifferentiated state. However, under differentiated conditions there appears to be closer alignment, particularly with respect to CD marker profile. These findings suggest that in most cases use of human PBMCs provides the researcher with the gold standard tool with which to study monocyte/macrophage function, especially for when investigating the role of immune cells in metabolic diseases. Nevertheless, monocyte-like cell lines will continue to have their place in research, especially as human blood is not always readily available and is invariably subject to a higher level of inherent variability across donors. Collectively this study indicates that although not perfect, in our hands the THP-1 cell line remains a simplistic surrogate for inflammation research and basic cell signalling mechanisms.

## Supporting information

S1 Table (**A**) Summary of the primers used in this study. (**B**) Summary of the antibodies used in this study.
(PDF)

S2 Table Raw Ct values used to calculate relative expression values (n = 3–10). PBMCs were differentiated using 10 ng/mL granulocyte-macrophage colony stimulating factor (GM-CSF) for 6 days to give M(GC) and activated using 100 ng/mL LPS and 20 μg/mL IFNγ for 24 h to generate M(GC)LPS/IFNγ. MCLCs were differentiated using 16 ng/mL phorbol-12-myristate-13-acetate (PMA) for 48 h. Grouped data ± SEM are shown (n = 3–10). An internal cutoff of >35 was used to determine if expression was observed. A selection of 35 genes were chosen that encode for inflammatory chemokines, cytokines, adipokines and their relevant receptors. These genes were chosen as they are associated with inflammation and have been implicated in the development and/or progression of obesity-induced insulin resistance. In addition, small subsets of genes encoding for regulatory factors and enzymatic processes that have been implicated in the pathogenesis of T2DM were profiled.
(PDF)

S3 Table Relative expression values used to generate Figs 1, 2A, 2B, 2C, 2D, 2E, 2F, 2G, 2H, 3A, 3B and 3C. PBMCs were differentiated using 10 ng/mL granulocyte-macrophage colony stimulating factor (GM-CSF) for 6 days to give M(GC) and activated using 100 ng/mL LPS and 20 μg/mL IFNγ for 24 h to generate M(GC)LPS/IFNγ. MCLCs were differentiated using 16 ng/mL phorbol-12-myristate-13-acetate (PMA) for 48 h. Grouped data ± SEM are shown (n = 3–10). Where no expression was detected the value was set to 0.0. A selection of 35 genes were chosen that encode for inflammatory chemokines, cytokines, adipokines and their relevant receptors. These genes were chosen as they are associated with inflammation and have been implicated in the development and/or progression of obesity-induced insulin resistance. In addition, small subsets of genes encoding for regulatory factors and enzymatic processes that have been implicated in the pathogenesis of T2DM were profiled.
(PDF)

## Abbreviations

PBMCs: Peripheral blood mononuclear cells
CD: cluster of differentiation
PMA: phorbol-12-myristate-13-acetate
FBS: fetal bovine serum
MCP-1: monocyte chemoattractant protein-1
