# Supplementary material for: Comparative genotypic and phenotypic analysis of human peripheral blood monocytes and surrogate monocyte-like cell lines commonly used in metabolic disease research
Source: PLoS One. 2018 May 10;13(5):e0197177. doi: 10.1371/journal.pone.0197177 (PMC5944989; doi:10.1371/journal.pone.0197177)
Supplement: S1 Table — (A) Summary of the primers used in this study. (B) Summary of the antibodies used in this study. (PDF) [file pone.0197177.s001.pdf]

Supplementary Table 1A - Summary of the primers used in this study

Class	Gene	Forward	Reverse
Housekeepers	*ACTB2*	CACCATTGGCAATGAGCGGTTC	AGGTCTTTGCGGATGTCCACGT
	GAPDH	GCACCGTCAAGGCTGAGAAC	ATGGTGGTGAAGACGCCAGT
Chemokines / cytokines	*CCL2*	TCTGTGCCTGCTGCTCATAG	GGGCATTGATTGCATCTGGC
	CCL3	ACTTTGAGACGAGCAGCCAGTG	TTTCTGGACCCACTCCTCACTG
	CCL4	GCTTCCTCGCAACTTTGTGGTAG	GGTCATACACGTACTCCTGGAC
	IL10	TCTCCGAGATGCCTTCAGCAGA	TCAGACAAGGCTTGGCAACCCA
	IL1β	GAAGCTGATGGCCCTAAACA	AAGCCCTTGCTGTAGTGGTG
	IL6	AGACAGCCACTCACCTCTTCAG	TTCTGCCAGTGCCTCTTTGCTG
	TGFβ	TACCTGAACCCGTGTTGCTCTC	GTTGCTGAGGTATCGCCAGGAA
	TNFα	CTCTTCTGCCTGCTGCACTTTG	ATGGGCTACAGGCTTGTCACTC
Receptors	*CCR1*	CAGAAAGCCCCAGAAACAAA	TCTCGTTCACCTTCTGGCAC
	CCR2	CAGGTGACAGAGACTCTTGGGA	GGCAATCCTACAGCCAAGAGCT
	CCR4	CACAGACCTTCCTCAGAGCC	TATCCGTGGGGTTCATTTTT
	CCR5	CATCCGTTCCCCTACAAGAA	GGCAGGGCTCCGATGTATAA
	FPR1	CCAGGAGACCCAGACCTAGA	GAGATGTTCGTGGGGAGAGA
	FPR2	TCCTCAGGAAAATGCACCAG	GCAGAACAGTGTAGCCAGCA
	FPR3	GCAGAACAGTGTAGCCAGCA	TCCACAGGAATCCAGGAACT
	IL10RA	GCCGAAAGAAGCTACCCAGTGT	GGTCCAAGTTCTTCAGCTCTGG
	IL10RB	GGAATGGAGTGAGCCTGTCTGT	AAACGCACCACAGCAAGGCGAA
	IL1R1	GTGCTTTGGTACAGGGATTCCTG	CACAGTCAGAGGTAGACCCTTC
	IL1R2	GGCTATTACCGCTGTGTCCTGA	GAGAAGCTGATATGGTCTTGAGG
	IL1RAP	CTGAGGATCTCAAGCGCAGCTA	AGCAGGACTGTGGCTCCAAAAC
	IL6R	GACTGTGCACTTGCTGGTGGAT	ACTTCCTCACCAAGAGCACAGC
	LTB4R1	AGGGATAGGCTACACCACCC	AAGATGTAGTGTTCATGGCCG
	LTB4R2	AAGGATGTCGGTCTGCTACCGT	CACACCACGAAGCCGTTGCCA
	TGFBR1	GACAACGTCAGGTTCTGGCTCA	CCGCCACTTTCCTCTCCAAACT
	TGFBR2	GTCTGTGGATGACCTGGCTAAC	GACATCGGTCTGCTTGAAGGAC
	TGFBR3	TGGAGTCTCCTCTGAATGGCTG	CCATTATCACCTGACTCCAGATC
	TLR4	CCCTGAGGCATTTAGGCAGCTA	AGGTAGAGAGGTGGCTTAGGCT
	TNFRSF1A	GGAGTGAGAGGCCATAGCTG	ATATTCCCACCAACAGCTCC
	TNFRSF1B	CGTTCTCCAACACGACTTCATCC	ACGTGCAGACTGCATCCATGCT
Enzymes	*IDO1*	GATGAAGAAGTGGGCTTTGC	CAGGCAGATGTTTAGCAATGA
	ITGAX	CAGCGGTACTGTACAAAGTTGG	ATCATTTCTCACTGGGCGGG
	KYNU	GAAAAGCATGCCCATACGAT	AGACCCCAGGGATTAACTGC
Regulatory factors	*IRF3*	ATGCACAGCAGGAGGATTTC	GTTGGCAGGTCTGGCTTATC
	IRF5	CAGAGCTCAGCTTGGTCCC	GATGGACTGGTTCATGGCAG
	IRF8	GAGGAGAAGAGCATGTTCCG	TTCCCTTTAAAAACTGCCCA

Supplementary Table 1B - Summary of the antibodies used in this study

Name	Supplier	Code
Purified mouse anti-human CD86	BD Biosciences	555655
Purified mouse anti-human CD11c	BD Biosciences	555391
Purified mouse anti-human CD80	BD Biosciences	557223
Purified mouse anti-human CD68	BD Biosciences	556059
Purified mouse anti-human CD14	BD Biosciences	555396
Purified mouse anti-human HLA-DR, DP, DQ	BD Biosciences	555557
Purified mouse anti-human CD163	BD Biosciences	556017
