# Supplementary material for: Comparative genotypic and phenotypic analysis of human peripheral blood monocytes and surrogate monocyte-like cell lines commonly used in metabolic disease research
Source: PLoS One. 2018 May 10;13(5):e0197177. doi: 10.1371/journal.pone.0197177 (PMC5944989; doi:10.1371/journal.pone.0197177)
Supplement: S2 Table — PBMCs were differentiated using 10 ng/mL granulocyte-macrophage colony stimulating factor (GM-CSF) for 6 days to give M(GC) and activated using 100 ng/mL LPS and 20 μg/mL IFNγ for 24 h to generate M(GC)LPS/IFNγ. MCLCs were differentiated using 16 ng/mL phorbol-12-myristate-13-acetate (PMA) for 48 h. Grouped data ± SEM are shown (n = 3–10). An internal cutoff of >35 was used to determine if expression was observed. A selection of 35 genes were chosen that encode for inflammatory chemokines, cytokines, adipokines and their relevant receptors. These genes were chosen as they are associated with inflammation and have been implicated in the development and/or progression of obesity-induced insulin resistance. In addition, small subsets of genes encoding for regulatory factors and enzymatic processes that have been implicated in the pathogenesis of T2DM were profiled. (PDF) [file pone.0197177.s002.pdf]

Supplementary Table 2. Ct values used to generate the relative expression values

Gene	PBMC	SEM	M(GC)	SEM	M(GC) LPS/IFNγ	SEM	U-937	SEM	U-937 + PMA	SEM	THP-1	SEM	THP-1 + PMA	SEM	HL-60	SEM	HL-60 + PMA	SEM
ACTB2	17.9	0.4	18.2	0.2	18.7	0.2	18.1	0.7	19.2	0.6	17.5	0.4	18.3	0.4	18.1	0.3	19.3	0.7
GAPDH	19.5	0.4	21.2	0.4	21.0	0.2	18.8	0.2	19.5	0.5	19.9	0.5	20.5	0.1	18.8	0.3	19.9	0.6
TNFα	24.8	0.8	25.0	0.2	22.3	0.3	30.3	2.1	27.9	0.9	29.4	1.2	30.8	1.3	27.5	1.4	27.5	0.8
TGFβ	22.6	0.1	22.8	0.5	19.2	0.9	24.1	0.8	24.2	0.9	24.4	0.9	21.4	0.9	23.4	0.6	23.5	0.9
IL6	29.8	0.1	31.2	1.3	27.0	0.2	37.3	1.4	36.2	3.0	37.7	2.1	38.0	1.9	38.4	2.1	36.9	2.0
IL10	32.0	0.0	26.6	0.3	26.7	0.3	37.6	1.9	32.1	0.9	39.2	1.7	38.9	1.0	36.5	1.6	38.7	1.2
IL1β	23.4	0.0	28.3	1.4	22.7	0.3	32.9	2.3	28.8	0.9	31.9	1.5	26.7	0.3	31.6	1.3	28.4	2.9
CCL2	25.6	0.1	24.7	0.1	23.2	0.3	26.6	2.0	24.0	0.9	27.8	1.9	27.0	1.1	25.3	1.0	27.3	1.1
CCL3	25.6	1.7	25.8	0.2	23.6	0.1	26.6	0.1	26.0	0.2	26.4	0.3	26.1	0.1	26.8	0.2	27.0	0.1
CCL4	29.6	0.8	29.6	0.2	25.0	0.2	38.3	0.5	34.6	0.3	35.7	4.2	32.8	0.1	38.5	1.9	34.6	0.8
ITGAX	23.2	0.1	19.2	0.0	18.9	0.2	33.3	2.5	26.8	0.4	27.3	0.8	24.1	0.1	30.9	2.1	23.7	1.0
IRF3	28.8	0.1	24.4	0.3	24.5	0.1	27.2	1.3	28.6	0.8	26.0	1.4	26.4	0.9	26.7	0.9	27.7	1.5
IRF5	27.0	0.3	22.9	0.6	23.5	0.1	27.5	0.7	27.8	0.9	27.2	1.4	29.3	0.1	28.1	1.8	30.8	1.1
IRF8	26.9	0.4	23.8	0.2	23.4	0.3	26.3	0.5	29.4	0.5	23.1	1.5	28.0	0.6	27.5	2.2	35.4	0.8
IDO1	35.5	0.4	34.8	2.2	25.4	0.1	40.0	0.0	39.6	0.6	40.0	0.1	40.0	0.0	40.0	0.0	40.0	0.0
KYNU	28.6	0.1	23.5	0.2	21.8	0.1	34.0	4.1	33.9	0.3	31.6	5.7	28.8	0.7	37.9	1.9	34.1	1.4
CCR1	26.5	1.6	24.6	0.2	24.2	0.3	31.4	2.3	29.0	0.1	27.6	0.2	28.4	0.1	29.9	0.1	28.8	0.0
CCR2	35.4	0.2	35.5	0.4	36.0	0.2	35.4	0.2	35.5	1.2	30.8	0.2	35.6	1.5	35.8	0.5	36.7	1.0
CCR4	37.5	1.9	33.9	0.3	36.2	1.3	39.6	0.6	39.0	1.7	33.6	0.7	40.0	0.0	40.0	0.0	39.4	1.0
CCR5	35.2	1.0	30.2	0.3	29.7	0.3	40.0	0.0	39.8	0.3	35.3	0.6	35.8	0.2	40.0	0.0	39.4	1.5
TNFRSF1A	35.5	0.1	35.2	0.2	34.0	0.5	31.5	1.3	33.2	0.4	34.3	1.0	34.3	0.9	36.0	3.6	35.4	0.8
TNFRSF1B	22.7	0.2	23.3	0.1	22.8	0.2	24.8	0.6	24.2	0.8	25.3	0.5	23.8	0.6	24.7	0.6	24.8	0.6
IL10RA	25.5	0.2	26.4	0.1	25.5	0.1	29.2	0.3	29.1	0.8	29.1	0.2	28.6	0.2	30.1	0.8	28.4	1.7
IL10RB	27.4	0.1	25.0	0.0	24.7	0.1	25.2	0.4	26.1	0.8	24.1	0.6	25.3	0.4	25.8	0.5	26.3	0.8
IL1R1	30.7	0.1	27.1	0.2	28.6	0.1	33.8	4.6	33.9	1.7	31.9	0.9	27.6	0.6	33.7	1.2	29.4	1.0
IL1R2	30.2	0.1	32.6	1.1	33.4	0.8	39.8	0.4	37.9	1.6	37.5	0.2	37.0	1.7	38.8	1.1	35.9	4.1
IL1RAP	31.9	0.1	31.0	0.2	31.9	0.4	29.5	0.7	31.0	1.0	28.9	0.8	30.5	0.5	28.4	0.2	30.2	0.5
TLR4	28.9	0.1	26.0	0.3	26.6	0.2	26.2	0.4	27.5	1.0	27.9	0.3	28.8	0.3	28.1	0.8	28.1	0.2
TGFBR1	29.0	0.1	24.8	0.3	25.5	0.1	24.1	0.5	24.6	0.9	24.1	0.7	25.3	0.5	25.9	0.1	26.5	0.4
TGFBR2	26.6	0.1	30.5	0.1	28.2	0.2	26.7	0.0	25.6	0.7	26.9	0.4	24.1	0.4	26.3	0.8	25.4	0.7
TGFBR3	34.4	0.2	34.5	1.3	37.3	1.6	27.4	0.2	31.1	0.2	28.0	0.5	29.4	0.6	38.1	0.4	39.3	0.8
IL6R	27.6	0.5	25.4	0.6	25.1	0.1	24.3	0.4	25.9	0.3	23.7	0.9	25.8	0.3	25.4	1.0	26.8	1.1
FPR1	23.9	0.8	28.6	0.1	27.7	0.1	35.3	0.6	32.1	0.2	34.7	0.4	35.6	0.5	33.0	0.3	36.1	0.8
FPR2	32.2	0.8	35.1	1.2	31.8	0.4	35.2	2.1	33.3	0.2	36.3	1.5	38.8	1.5	33.8	1.0	39.4	1.4
FPR3	35.4	0.6	27.0	0.3	25.8	0.1	40.0	0.0	40.0	0.0	40.0	0.0	40.0	0.0	40.0	0.0	40.0	0.0
LTB4R1	26.3	0.4	28.3	0.1	29.2	0.2	29.2	0.1	30.7	0.2	28.8	0.2	29.2	0.3	29.7	0.1	30.8	0.5
LTB4R2	26.8	0.1	29.9	0.2	30.4	0.3	25.3	0.1	27.5	0.2	27.8	0.1	29.3	0.1	26.7	0.1	29.3	0.1

PBMCs were differentiated using 10 ng/mL granulocyte-macrophage colony stimulating factor (GM-CSF) for 6 days to give M(GC) and activated using 100 ng/mL LPS and 20 µg/mL IFN for 24 h to generate M(GC/LPS/IFN).
MCLCs were differentiated using 16 ng/mL phorbol-12-myristate-13-acetate (PMA) for 48 h. Grouped data ± SEM are shown (n=3-10). An internal cutoff of >35 was used to determine if expression was observed
A selection of 35 genes were chosen that encode for inflammatory chemokines, cytokines, adipokines and their relevant receptors.
These genes were chosen as they are associated with inflammation and have been implicated in the development and/or progression of obesity-induced insulin resistance. In addition, small subsets of genes encoding for regulatory factors and enzymatic processes that have been implicated in the pathogenesis of T2DM were profiled.
