# Supplementary material for: Comparative genotypic and phenotypic analysis of human peripheral blood monocytes and surrogate monocyte-like cell lines commonly used in metabolic disease research
Source: PLoS One. 2018 May 10;13(5):e0197177. doi: 10.1371/journal.pone.0197177 (PMC5944989; doi:10.1371/journal.pone.0197177)
Supplement: S3 Table — PBMCs were differentiated using 10 ng/mL granulocyte-macrophage colony stimulating factor (GM-CSF) for 6 days to give M(GC) and activated using 100 ng/mL LPS and 20 μg/mL IFNγ for 24 h to generate M(GC)LPS/IFNγ. MCLCs were differentiated using 16 ng/mL phorbol-12-myristate-13-acetate (PMA) for 48 h. Grouped data ± SEM are shown (n = 3–10). Where no expression was detected the value was set to 0.0. A selection of 35 genes were chosen that encode for inflammatory chemokines, cytokines, adipokines and their relevant receptors. These genes were chosen as they are associated with inflammation and have been implicated in the development and/or progression of obesity-induced insulin resistance. In addition, small subsets of genes encoding for regulatory factors and enzymatic processes that have been implicated in the pathogenesis of T2DM were profiled. (PDF) [file pone.0197177.s003.pdf]

Supplementary Table 3. Relative expression values used to generate Figure 1

Gene	PBMC	SEM	M(GC)	SEM	M(GC) LPS/IFNγ	SEM	U-937	SEM	U-937 + PMA	SEM	THP-1	SEM	THP-1 + PMA	SEM	HL-60	SEM	HL-60 + PMA	SEM
TNFα	1.0	0.2	0.8	0.1	2.9	0.6	1.0	0.2	11.0	2.1	1.0	0.2	1.0	0.3	1.0	0.2	1.5	0.3
TGFβ	1.0	0.1	0.7	0.1	1.1	0.1	1.0	0.2	1.3	0.2	1.0	0.2	8.4	1.8	1.0	0.3	2.3	0.2
IL6	1.0	0.6	3.7	0.7	40.1	8.1	0.0	0.0	0.0	0.0	0.0	0.0	0.0	0.0	1.0	0.4	6.3	0.8
IL10	1.0	0.6	1.2	0.4	0.8	0.1	1.0	0.5	36.7	18.6	0.0	0.0	0.0	0.0	1.0	0.5	0.4	0.1
IL1β	1.0	0.4	2.1	1.3	4.3	0.7	0.0	0.0	1.1	0.2	1.0	0.5	52.4	10.6	1.0	0.6	11.8	3.7
CCL2	1.0	0.2	10.3	2.9	35.3	10.0	1.0	0.3	17.1	6.2	1.0	0.6	1.9	0.4	1.0	0.1	0.7	0.2
CCL3	1.0	0.5	1.0	0.1	3.6	0.6	1.0	0.2	2.9	0.7	1.0	0.2	1.9	0.3	1.0	0.2	1.1	0.2
CCL4	1.0	0.4	1.7	0.2	22.5	5.2	1.0	0.3	17.1	4.9	1.0	0.4	6.5	1.9	1.0	0.6	7.2	2.8
ITGAX	1.0	0.1	7.6	2.2	5.1	0.4	0.0	0.0	0.7	0.2	1.0	0.4	16.3	2.7	0.0	0.0	4.4	0.6
IRF3	1.0	0.3	2.2	0.2	2.8	0.3	1.0	0.1	1.0	0.2	1.0	0.1	1.6	0.3	1.0	0.1	1.3	0.2
IRF5	1.0	0.2	3.1	1.0	3.4	0.6	1.0	0.2	2.0	0.6	1.0	0.2	0.8	0.2	1.0	0.1	0.9	0.2
IRF8	1.0	0.1	1.2	0.1	1.9	0.2	1.0	0.2	0.3	0.1	1.0	0.2	0.2	0.1	1.0	0.1	0.0	0.0
IDO1	0.0	0.0	0.0	0.0	2.1	0.5	0.0	0.0	0.0	0.0	0.0	0.0	0.0	0.0	0.0	0.0	0.0	0.0
KYNU	1.0	0.4	5.1	0.4	20.1	2.7	1.0	0.3	1.3	0.4	1.0	0.4	2.0	0.4	0.0	0.0	0.4	0.1
CCR1	1.0	0.4	1.9	0.1	3.2	0.6	1.0	0.2	8.9	1.3	1.0	0.3	1.2	0.3	1.0	0.2	2.9	0.4
CCR2	0.0	0.0	0.0	0.0	0.0	0.0	0.0	0.0	0.0	0.0	1.0	0.2	0.2	0.1	0.0	0.0	0.0	0.0
CCR4	1.0	0.1	2.9	0.4	1.4	0.6	0.0	0.0	0.0	0.0	1.0	0.2	0.0	0.0	0.0	0.0	0.0	0.0
CCR5	1.0	0.2	8.5	1.0	12.3	2.6	0.0	0.0	0.0	0.0	0.0	0.0	0.0	0.0	0.0	0.0	0.0	0.0
TNFRSF1A	1.0	0.2	1.0	0.1	1.9	0.4	1.0	0.2	0.6	0.2	1.0	0.3	1.8	0.3	0.0	0.0	0.0	0.0
TNFRSF1B	1.0	0.1	0.6	0.0	0.9	0.1	1.0	0.1	3.1	0.7	1.0	0.1	4.2	0.9	1.0	0.2	1.4	0.2
IL10RA	1.0	0.1	0.6	0.1	1.3	0.2	1.0	0.1	2.9	0.6	1.0	0.2	2.5	0.5	1.0	0.2	5.0	1.2
IL10RB	1.0	0.1	1.9	0.1	3.0	0.4	1.0	0.1	1.3	0.2	1.0	0.2	0.9	0.2	1.0	0.2	1.2	0.2
IL1R1	1.0	0.2	3.3	0.6	2.6	0.8	0.0	0.0	0.0	0.0	1.0	0.4	29.6	8.6	1.0	0.5	15.5	3.4
IL1R2	1.0	0.2	0.3	0.0	0.4	0.1	0.0	0.0	0.0	0.0	0.0	0.0	0.0	0.0	0.0	0.0	0.0	0.0
IL1RAP	1.0	0.1	0.8	0.1	0.5	0.1	1.0	0.1	0.9	0.2	1.0	0.2	0.6	0.1	1.0	0.2	0.6	0.2
TLR4	1.0	0.1	2.3	0.2	1.9	0.2	1.0	0.1	0.9	0.2	1.0	0.2	1.0	0.2	1.0	0.2	1.5	0.2
TGFBR1	1.0	0.2	5.2	0.5	3.9	0.5	1.0	0.2	1.9	0.4	1.0	0.2	1.0	0.2	1.0	0.2	1.2	0.2
TGFBR2	1.0	0.1	2.0	0.1	1.3	0.2	1.0	0.1	3.3	0.8	1.0	0.2	4.7	1.2	1.0	0.2	2.1	0.4
TGFBR3	0.0	0.0	0.0	0.0	0.0	0.0	1.0	0.1	0.2	0.0	1.0	0.2	0.7	0.2	0.0	0.0	0.0	0.0
IL6R	1.0	0.1	1.3	0.1	2.0	0.2	1.0	0.1	0.9	0.1	1.0	0.2	0.5	0.1	1.0	0.2	0.7	0.1
FPR1	1.0	0.2	0.3	0.1	0.4	0.1	1.0	0.2	9.8	1.1	0.0	0.0	0.0	0.0	1.0	0.2	0.4	0.2
FPR2	1.0	0.3	8.2	5.3	3.1	0.7	1.0	0.6	7.6	4.5	0.0	0.0	0.0	0.0	1.0	0.5	0.1	0.0
FPR3	0.0	0.0	0.8	0.1	1.5	0.3	0.0	0.0	0.0	0.0	0.0	0.0	0.0	0.0	1.0	0.1	1.4	0.2
LTB4R1	1.0	0.2	0.4	0.1	0.3	0.0	1.0	0.1	1.0	0.3	1.0	0.2	1.2	0.3	1.0	0.1	0.8	0.3
LTB4R2	1.0	0.2	0.5	0.1	0.4	0.1	1.0	0.1	0.5	0.0	1.0	0.2	0.7	0.2	1.0	0.1	0.3	0.0

PBMCs were differentiated using 10 ng/mL granulocyte-macrophage colony stimulating factor (GM-CSF) for 6 days to give M(GC) and activated using 100 ng/mL LPS and 20 μg/mL IFN for 24 h to generate M(GC/LPS/IFN).
MCLCs were differentiated using 16 ng/mL phorbol-12-myristate-13-acetate (PMA) for 48 h. Grouped data ± SEM are shown (n=3-10). Where no expression was detected the value was set to 0.0
A selection of 35 genes were chosen that encode for inflammatory chemokines, cytokines, adipokines and their relevant receptors.
These genes were chosen as they are associated with inflammation and have been implicated in the development and/or progression of obesity-induced insulin resistance. In addition, small subsets of genes encoding for regulatory factors and enzymatic processes that have been implicated in the pathogenesis of T2DM were profiled.
